# Microspinning: Local Surface Mixing via Rotation of Magnetic Microparticles for Efficient Small-Volume Bioassays

**DOI:** 10.3390/mi11020175

**Published:** 2020-02-07

**Authors:** Su Deok Kim, Seo Woo Song, Dong Yoon Oh, Amos Chungwon Lee, Jeong Woo Koo, Taehun Kang, Min Chang Kim, Changhee Lee, Yunjin Jeong, Hyun Yong Jeong, Daewon Lee, Seongkyu Cho, Sunghoon Kwon, Jiyun Kim

**Affiliations:** 1 Institutes of Entrepreneurial BioConvergence, Seoul National University, Seoul 08826, Korea; ksd9789@gmail.com (S.D.K.); ssw0313@gmail.com (S.W.S.); amoslee89@gmail.com (A.C.L.); chlee6067@gmail.com (C.L.); jeancompany09@gmail.com (Y.J.); cho61@snu.ac.kr (S.C.); 2 Department of Electrical and Computer Engineering, Seoul National University, Seoul 08826, Korea; agongee@snu.ac.kr; 3 Interdisciplinary Program for Bioengineering, Seoul National University, Seoul 08826, Korea; dyoon.oh@gmail.com; 4 Department of Material Science and Engineering, Seoul National University, Seoul 08826, Korea; koopoe1230@snu.ac.kr; 5 School of Environmental Engineering, University of Seoul, Seoul 08826, Korea; 6 BK21+ Creative Research Engineer Development for IT, Seoul National University, Seoul 08826, Korea; hyunyong.jeong1@gmail.com (H.Y.J.); powerballx5@gmail.com (D.L.); 7 School of Materials Science and Engineering, Ulsan National Institute of Science and Technology, Ulsan 44919, Korea

**Keywords:** microparticle, micromixing, magnetic particle, magnetization patterning, suspension array

## Abstract

The need for high-throughput screening has led to the miniaturization of the reaction volume of the chamber in bioassays. As the reactor gets smaller, surface tension dominates the gravitational or inertial force, and mixing efficiency decreases in small-scale reactions. Because passive mixing by simple diffusion in tens of microliter-scale volumes takes a long time, active mixing is needed. Here, we report an efficient micromixing method using magnetically rotating microparticles with patterned magnetization induced by magnetic nanoparticle chains. Because the microparticles have magnetization patterning due to fabrication with magnetic nanoparticle chains, the microparticles can rotate along the external rotating magnetic field, causing micromixing. We validated the reaction efficiency by comparing this micromixing method with other mixing methods such as simple diffusion and the use of a rocking shaker at various working volumes. This method has the potential to be widely utilized in suspension assay technology as an efficient mixing strategy.

## 1. Introduction

With the advantages of fast reaction time and low reagent cost, miniaturization has become a general trend in the field of chemical and biological research, and has been utilized in existing bioassays, separation technologies, and chemical synthesis techniques [1,2,3]. As the reaction volume decreases, however, surface tension becomes dominant. Thus, common mixing methods in macroscale reactions, such as vortexing or rocking, are not as effective on the microscale [1,3]. Concerning the microfluidic channel platform, one of the main miniaturization approaches, reagent mixing, is implemented by channel structures that induce chaotic advection, or active methods such as micropump or acoustic mixing [4,5,6,7,8]. As another miniaturization approach, microarray-based techniques are broadly used in many types of research [9]. They can be categorized into spatial microarrays and suspension microarrays (in other words, a bead-based platform). The suspension array is a microarray format with greater flexibility than positional arrays in terms of homogeneous reaction and multiplexity [9]. However, micromixing techniques for microfluidic platform are mostly not compatible with the bead-based suspension array platform because of their dissimilarity. Many commercialized suspension array technologies use conventional well plates because of their user-friendliness.

Magnetic methods, on the other hand, are accessible in a remotely working manner and have been widely adopted in many applications such as biochemical assays and microactuators [10,11,12,13]. One commonly used actuating technique utilizing magnetic properties in chemical and biological fields involves the use of Dynabead^®^ superparamagnetic beads (SPBs), which are uniform polystyrene spherical beads that have been made magnetizable, for magnetic separation of many biological materials [14,15]. However, this bead-based technique only utilizes the feature of magnetic attraction. Despite the wide usage of magnetic beads, as miniaturization of biological assays proceeds, mixing on the microscale in a microwell is still difficult because of the small dimensions and strong viscous forces. 

Here, we propose a new active mixing method for bead-based suspension array technology that exploits magnetic particle rotation under an external magnetic field. Under the external magnetic field, magnetic nanoparticles in a polymer resin are self-assembled to form chainlike structures and create magnetization [10]. After polymerization, this programmed magnetization by self-assembled magnetic nanoparticle chains within the microparticle makes the microparticles spin under the external rotating magnetic field [11]. The magnetic microparticle rotation induces homogeneous microscale mixing around the substrate in the solution. Although there have been a few attempts at utilizing chains of magnetic particles under an external magnetic field as a fluidic mixing strategy, no other approaches have demonstrated that the magnetic nanoparticle chain inside shape-encoded microparticles enables efficient bioassay under an external rotating magnetic field as a multiplexed suspension array [16,17,18,19,20]. In this study, we compared the mixing efficiency of three methods (simple diffusion, rocking, and particle spinning) by quantifying the amount of reaction in different-sized well plates (24-well, 96-well, and 384-well) over a given period of time. We verified that the magnetic particle spinning shows the highest reaction efficiency regardless of reaction volume among all experimental methods of mixing. Furthermore, two possible mechanisms behind the enhanced assay efficiency are discussed. First, the magnetic microparticles sedimentation in solution by density can be overcome by magnetic rotation. Second, the rotation of the microparticle causes local surface mixing of the solution near the microparticle. Moreover, the smaller the volume of the reaction well, the greater the difference in reaction efficiency. Therefore, we expect that our technique will result in great advances in microscale mixing utilized in suspension array-based studies of biological systems.

## 2. Methods and Materials

### 2.1. Fabrication of Biotinylated Magnetic Microparticle

We used biotinylated microparticles with 1-dimensional (1D) magnetic nanoparticle chains. These biotinylated microparticles were fabricated via a conventional photolithography technique. The prepolymer solution was prepared by mixing 79% (v/v) poly(ethylene glycol) diacrylate (PEG-DA) (M_n_ = 700; Sigma-Aldrich), 4% (v/v) photoinitiator (2-hydroxy-2-methylpropiophenone 97%, Sigma-Aldrich), 17% (v/v) Deionized (D.I) water, 8 mg/mL Acrylate-PEG-Biotin (PEG M_n_ = 2000; CreativePEGWorks, Durham, NC, USA), and 4 mg/mL Fe_3_O_4_ nanoparticles (50 nm; Sigma-Aldrich, St. Louis, MO, USA). Here, Fe_3_O_4_ magnetic nanoparticles were used to make self-assembled 1D magnetic nanoparticle chains for magnetization patterning. Then, 15,225 microparticles with 1D magnetic chains were polymerized simultaneously with 5 s exposure of UV with a 18.6 mW/cm^2^ intensity, under a 3000 Gauss neodymium magnet. The microparticles were 100 µm in diameter and 70 µm in height (more information described in Supplementary Material Figures S1–S5). The fabricated microparticles were stored in 1% bovine serum albumin (BSA)—phosphate buffered saline (PBS) solution to block the reactive functional group and to prevent nonspecific binding.

### 2.2. Biotin–Streptavidin-R-phycoerythrin Conjugate SAPE Assay Method

In order to validate the enhancement of reaction speed through micromixing, a biotin–streptavidin binding assay was performed with biotin-conjugated microparticles in Streptavidin-R-phycoerythrin conjugate (SAPE, Invitrogen, Carlsbad, CA, USA) solution. The stronger the fluorescence intensity of the microparticles after a certain incubation time, the more molecular binding occurred. In this study, incubation was performed for 30 min at room temperature in 1 µg/mL SAPE in 0.1% BSA–PBS solution. Reactions were performed in a multiwell plate (24-well, 96-well, and 384-well plates at reaction volumes of 400 µL, 100 µL, and 25 µL, respectively). Then, 50 to 100 microparticles were added in a single reaction chamber. After incubation, microparticles were moved into a 1.5 mL test tube and washed 4 times with 0.5% tween and 1% BSA–PBS solution using a magnetic separation stand. As will be described in detail later, the reaction efficiencies of different mixing methods in different reaction volumes were compared.

### 2.3. Data Analysis

After the washing step, all the microparticles were collected in a single microwell of a 96-well plate, and fluorescence images of microparticles in a whole microwell area were captured using automated fluorescence microscopy (Nikon Digital Sight DS-Ri1, Nikon C-LHGFI HG LAMP, Tokyo, Japan). Using a circle detection algorithm in ImageJ, the average pixel intensities of every microparticle were obtained. The fluorescence intensity data are presented in a bar graph, and error bars represent standard deviation. 

## 3. Results

### 3.1. The Principle and the Fabrication of Magnetically Rotating Microparticles with Magnetization Patterning

**Figure 1:**
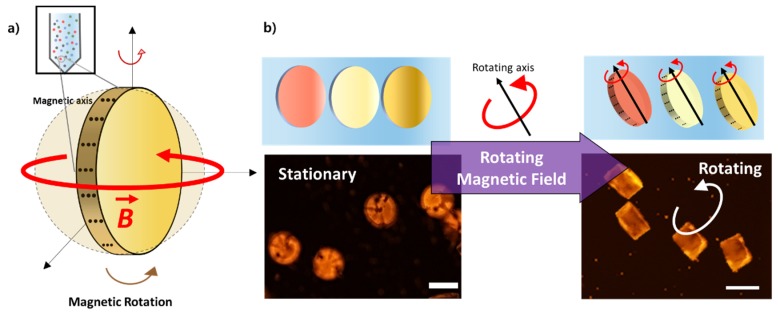
Patterned magnetization of polymer microparticles enables efficient microscale mixing. (**a**) A schematic of a microparticle embedded with aligned magnetic nanoparticles and its rotation. (**b**) 3D schematic and fluorescence micrograph of efficient mixing under rotating magnetic field. Scale bar, 100 µm.

**Figure 2:**
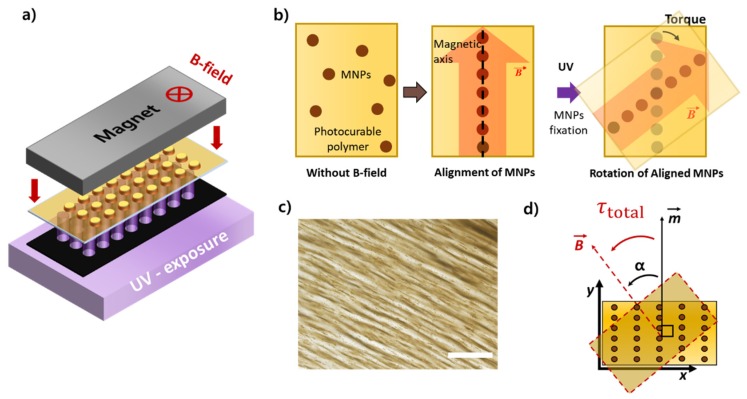
(**a**) Fabrication using conventional lithography setup. UV light is exposed to the dynamic photomask and then reflected to the substrate with photocurable polymers on an automated stage. (**b**) Self-assembly of magnetic nanoparticles inside polymer resin along the uniform magnetic field line. (**c**) Brightfield micrograph of the 1D magnetic chain structure located in the cross-section of the microparticle. Scale bar, 100 µm. (**d**) A schematic of torque induction and the particle spinning based on 1D magnetic nanoparticle chain structures.

The main advantage of using magnetic microparticles with 1D magnetic chains over conventional magnetic beads is the use of programmable magnetization for microspinning the particles (Figure 1a). By controlling the self-assembled pattern of magnetic nanoparticles during the fabrication process, the magnetization direction and intensity can be programmed in the polymeric microparticle (Figure 2b). If we apply an external magnetic field to the microparticle, the interaction between 1D magnetic chains and the external magnetic field results in the application of magnetic torque to the microparticles (Figure 2c,d). The torque exerted on a single chain in the polymeric microparticle was found to be τ=m ×B, where τ is the magnetic torque, ***m*** is the magnetic moment of an average 1D chain, and ***B*** is the magnetic flux density of the applied uniform magnetic field. The total torque applied on a polymeric microparticle embedded with *N* number of chains under the external magnetic field is expected to be *N* times the torque on a single chain.

The polymeric microparticles embedded with 1D magnetic chains were fabricated via conventional photolithography techniques (Figure 2a). Biotin molecules are covalently conjugated with polymer matrix by a poly(ethylene glycol)-acrylate (PEG-acrylate) functional group. Magnetic nanoparticles were used to program the magnetization in the polymer structure. Without an external magnetic field, magnetic nanoparticles are uniformly dispersed in the photocurable polymer resin. Before the photopolymerization, we applied an external magnetic field to form self-assembled chainlike structures of magnetic nanoparticles (Figure 2b). When polymer resin is polymerized by UV exposure under this condition, the self-assembled pattern of magnetic nanoparticles is fixed in the polymer matrix implanting the magnetization, and the resulting microparticles can induce magnetic torque when the magnetic field is applied. 

In addition, these polymeric microparticles are designed to have the shape code on their surface for use in multiplexed bioassays (Supplementary Figure S2). Multiplexed bioassays using shape-encoded microparticles enable the simultaneous detection and quantitation of multiple secreted proteins in a single microwell [21,22,23]. Thus, shape-encoded microparticles with 1D chains of magnetic nanoparticles are capable of using both magnetic attraction and magnetic rotating, which can result in diverse functional advantages in comprehensive bioassays (Figure 1b).

### 3.2. Reaction Performance of Magnetically Rotating Microparticles

**Figure 3:**
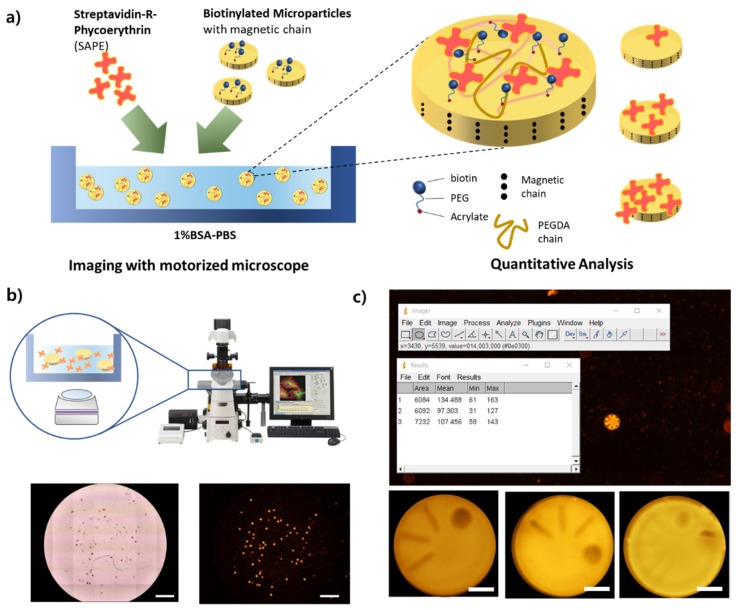
The design of a binding assay for validation and its analysis process. (**a**) A schematic of the validation method using binding between the biotinylated microparticle and streptavidin with fluorophore as a proof-of-concept. (**b**) The process of data acquisition using fluorescence microscopy with a motorized stage. Scale bar, 1 mm. (**c**) Quantitative analysis based on fluorescence image acquired after large-scale imaging. Scale bar, 25 µm.

To verify whether micromixing by the spinning of magnetic microparticles increases the reaction efficiency in bioassays, we designed a simple binding assay that is able to measure interactions between two molecules, such as a protein binding to another protein, for the proof-of-concept of our hypothesis (Figure 3a). The binding assay process and the comparative results of mixing performance are described in this section. The basic binding assay that we adopted for our demonstration used the principle of binding biotin with streptavidin. Polymeric microparticles have biotins in their polymer chain, and streptavidin is covalently attached with R-phycoerythrin as a fluorescent label. In this paper, streptavidin-R-phycoerythrin conjugate (SAPE) was used to quantify the reaction efficiency by measuring the fluorescence intensity after incubation for the same amount of analytes at a given period of time (Figure 3b,c). 

We compared three different mixing methods to verify the effect of the micromixing technique using the remote microspinning of magnetic microparticles: (i) the microspinning of magnetic microparticles under the rotating magnetic field; (ii) rocking of the entire well plate using a shaker; and (iii) simple diffusion leaving the plate stationary at room temperature (Supplementary Figure S3). These experiments were repeatedly performed using 24-, 96-, and 384-well plates.

**Figure 4:**
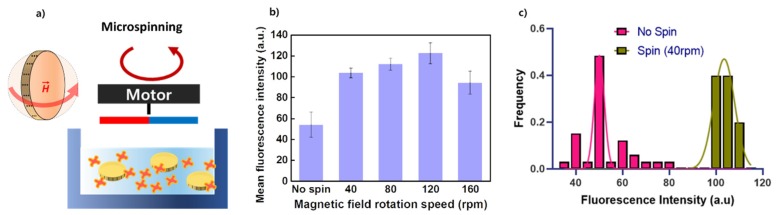
The optimization of magnetic field rotation speed. (**a**) A schematic of micromixing by microparticle spinning under a rotating magnetic field (**b**) The reaction efficiency was quantitatively measured to determine the appropriate conditions for the rotating speed of the external rotating magnetic field. (**c**) The distribution of fluorescence intensity of microparticles from the dataset between the groups of “No spin” and “40 rpm” from (**b**).

To optimize the micromixing effect of spinning magnetic microparticles, we firstly investigated the rotation speed of the magnetic particles, which shows the maximum binding efficiency in the assay (Figure 4a). As shown in Figure 4b, as the microspinning speed increased, the binding efficiency increased until it peaked at 120 rpm of the rotating magnet and then decreased. Generally, the magnetic microparticle exhibits a rotation in perfect accordance with the angular velocity of the magnetic field when the magnetic torque and the hydrodynamic drag force reaches a counterbalance. Considering the fact that the effect of micromixing peaked around 120 rpm of magnets, the spinning of magnetic microparticles failed to follow the speed of the external magnetic field due to the resistance from the surrounding viscous solution. Additionally, the rpm of the rocking shaker was determined to be 100, the fastest in the speed range according to the equipment manual.

**Figure 5:**
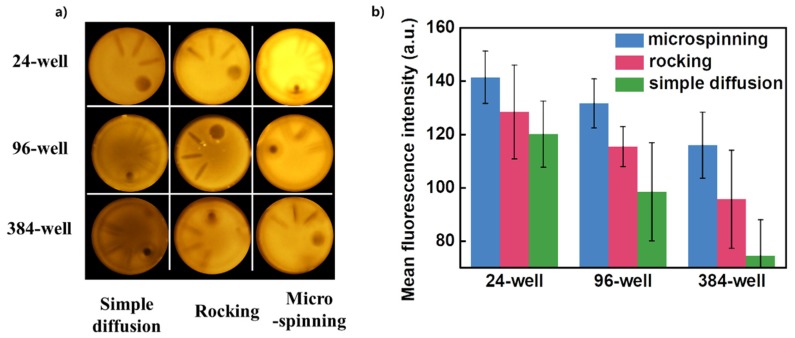
The comparative results of bioassay performance using a variety of mixing methods. (**a**) Fluorescence images of the representative bead image from each experimental group. (**b**) Quantitative analysis of reaction efficiency based on fluorescence intensity from each experiment.

Under this optimized condition, we compared the results of binding assays in three different methods using 24-well, 96-well, and 384-well plates. When micromixing was compared with simple diffusion, the reaction efficiency of micromixing was improved by 17%, 33%, and 55% in 24-well, 96-well, and 384-well plates, respectively. In addition, when micromixing was compared with the rocking method, the efficiency was shown to have improved by 10%, 14%, and 21% in the wells of the 24-well, 96-well, and 384-well plates, respectively. On the basis of these results, we confirmed that as the size of the reaction chamber becomes miniaturized, the micromixing by substrate microspinning becomes more effective compared to conventional mixing methods (Figure 5b).

## 4. Discussion

In summary, we propose a new active mixing method for microparticle-based suspension array technology (SAT) that exploits magnetic particle rotation under an external magnetic field. Under the external magnetic field, magnetic nanoparticles in a polymer resin are self-assembled to form chainlike structures and create the magnetization. After polymerization, this programmed magnetization by self-assembled magnetic nanoparticle chains within the microparticle makes the microparticles spin under the external rotating magnetic field. Unlike many previous magnetic mixing method for enhanced fluidic mixing in low Reynolds number flows (Re ≤ 1) and in stagnant fluids, our approach differs by having the following advantages: it integrates using encoded magnetic microparticles for multiplexed bioassays and magnetic actuation for efficient mixing at the same time [16,17,18,19,20]. With the proposed technology, we have shown that magnetically rotating the microparticle for the bioassay increased reaction efficiency. 

One of the possible mechanisms for the increased efficiency can be explained in terms of the increased exposure of the available binding sites in microparticles to the assay solution. The two-fold difference in the fluorescence intensities between the group with and without “spin” seems to be caused by the increased exposure of both sides of the microparticle to the small molecules (Figure 4b). Furthermore, we identified that the rotation of microparticle around the vertical axis causes a more uniform reaction than the stationary state of the microparticle. This is shown in the lower standard deviation between the group with and without the "spin" (Figure 4c)

The increased efficiency of the bioassay can also be explained by the effect of local fluid flow induced by the local surface agitation under an external magnetic rotating field. The rotating magnetic particle can induce local fluid near the rotating particle to cause fluid flow outward to achieve local surface agitation at a low Reynolds number. The polymeric microparticle used for our experiment (D = 100 µm, H = 80 µm) had a disk shape. When it rotated along the z-axis, as shown in Figure 1a, the flow field around the 3D contour shape of the rotating particle can be approximated by the flow around a rotating sphere. On the basis of the creeping flow around a rotating sphere, we conducted a simulation analysis.

To simply simulate the flow, the flow field of the rotating sphere can be expressed in Cartesian coordinates as follows [24]:

(1) $$f\left(x,y\right)=\;\frac{y^{2}\left(-\frac{25}{8}+\frac{25}{4\sqrt{x^{2}+y^{2}}}-\frac{25}{8\left(x^{2}+y^{2}\right)}\right)}{x^{2}{\left(\frac{y^{2}}{x^{2}}+1\right)}^{3/2}}$$

Equation (1) is a simplified version of the two-dimensional flow field for a rotating sphere at a low Reynolds number showing the first order solutions. In the derivation of this equation, a regular perturbation expansion is used. At the lowest order, inertial terms are neglected, and the flow is one of pure rotation that decays away from the sphere. The perturbation improvement takes weak inertia into account and this causes a secondary flow in the r direction.

Considering the contour plot and visualized streamline of the flow field around the rotating sphere derived from Equation (1), respectively, we could identify that the flow shows a circulation pattern. Flows are strongest outward along the equator, meaning the x–y plane in this case, and this fluid is replaced by inflows from a rotational axis, the z-axis in this case (Supplementary Figures S4 and S5). With this simulated streamline for a single rotating sphere at a low Reynolds number, we could determine that the streamlines around the rotating sphere spread out from the rotating axis [24].

Importantly, streamlines should not remain at a fixed distance from the center of the microparticle. Rather, streamlines should be dispersed over the radial distance of the microparticle by inducing local mass transport for efficient mixing. From our simulated results, the streamlines around the rotating particle are not restricted to a narrow distance from the rotating axis of the microparticle, but instead are heading outward as suggested above. Generally, when the streamline fields are as complex and entangled as possible, the local mixing tends to be produced well [25,26,27]. However, although the simulation result showed the strong local flow heading outward around the rotating sphere, it does not guarantee that the microparticle can generate whole-fluid mixing, since the fluid mass involved in the secondary flow may be so small that they hardly impact the whole fluid. 

Although this platform presents a proof-of-concept for micromixing using microparticles, there is more work that needs to be done before the platform can be extensively use. One of the main advantages of this platform is the generality of the platform’s application. For most of the microparticles that are fabricated through polymerization, the aligned magnetic nanoparticles can simply be incorporated during the fabrication process. This integration will accelerate the efficiency of bioassays utilizing microparticles. For example, if this platform can be integrated to a point-of-care (POC) study utilizing encoded microparticles, the multiplexed colorimetric POC autoantibody detection will achieve a faster reaction in a reduced time [23].

## 5. Conclusions

We have shown that this polymeric microparticle embedded with magnetic nanoparticle chains could increase the reaction efficiency when performing suspension array-based assays in stagnant fluids such as in conventional well plates. Common mixing methods, such as vortexing or rocking, are not as effective on the microscale, mainly because surface tension dominates microscopic scale environments with a low Reynolds number. We developed magnetically actuated microparticles with programmed magnetization to achieve an efficient reaction. Because the microparticle has the magnetization programmed by magnetic nanoparticle chains within a polymer matrix, it can rotate along the external magnetic field, resulting in local surface agitation in the fluidic reaction environment. This magnetic microparticle technology not only supports the magnetic pulling method for medium exchange or separation in suspension array-based bioassays, but also increases reaction efficiency by rotating microparticles in the surrounding medium. We believe that this microparticle with 1D magnetic chains will be a universal and versatile active substrate with the potential to be widely adopted in current magnetic bead-based biochemical assays.

## Supplementary Materials

The following are available online at https://www.mdpi.com/2072-666X/11/2/175/s1, Figure S1. Chemical process of the preparation of polymerized microparticles for biochemical binding assay, Figure S2. A variety of shape-encoded microparticle for multiplexed bioassay, Figure S3. A schematic illustration of the comparison between experimental setup and detailed condition for experiment, Figure S4. A contour plot of the creeping flow field around the rotating sphere in low Reynolds number, Figure S5. A visualized streamline of flow field around the rotating sphere.
