# Supplementary material for: Microspinning: Local Surface Mixing via Rotation of Magnetic Microparticles for Efficient Small-Volume Bioassays
Source: Micromachines (Basel). 2020 Feb 7;11(2):175. doi: 10.3390/mi11020175 (PMC7074623; doi:10.3390/mi11020175)
Supplement: Supplementary file 1 [file micromachines-11-00175-s001.pdf]

micromachines

Supplementary Materials: Microspinning: Local Surface Mixing via Rotation of Magnetic Microparticles for Efficient Small-Volume Bioassays

Su Deok Kim, Seo Woo Song, Dong Yoon Oh, Amos Chungwon Lee, Jeong Woo Koo, Taehun Kang, Min Chang Kim, Changhee Lee, Yunjin Jeong, Hyun Yong Jeong, Daewon Lee, Seongkyu Cho, Sunghoon Kwon and Jiyun Kim

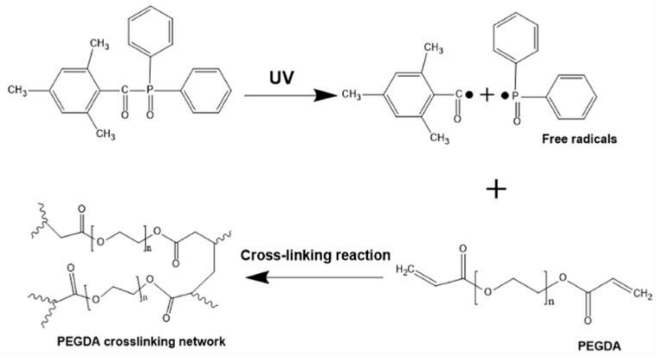

PEG-DA + photoinitiator crosslinking

Pre-polymer Solution
PEG-DA : 79% (v/v)
DAROCUR 1173 : 4% (v/v)
Acrylate-PEG-Biotin : 8mg/ml
Fe_3O_4 : 4mg/ml
D.I. water : 17% (v/v)

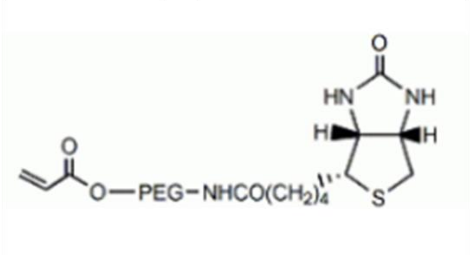

Acrylate PEG Biotin

Figure S1. Chemical process of the preparation of polymerized microparticles for biochemical binding assay.

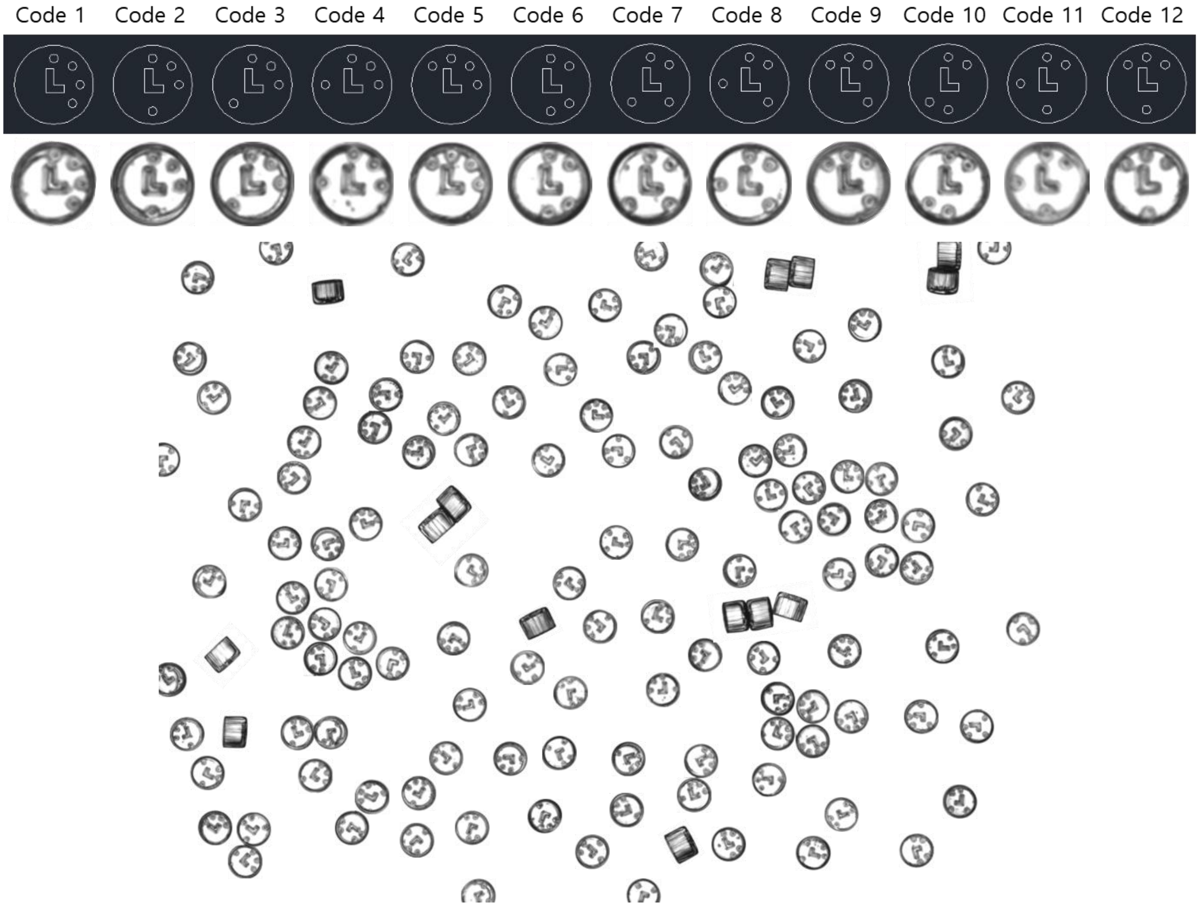

Figure S2. A variety of shape-encoded microparticle for multiplexed bioassay.

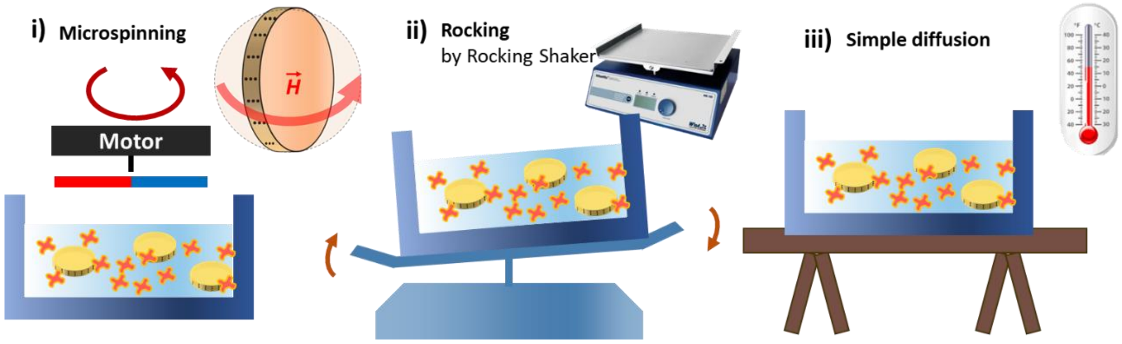

Figure S3. A schematic illustration of the comparison between experimental setup and detailed condition for experiment. Three different mixing methods such as i) Micromixing by microparticle spinning under rotating magnetic field ii) Rocking by Rocking Shaker iii) Simple diffusion at R.T. Each binding assay was done for 30 min, the RPM of the external rotating field was 120 RPM, and the RPM of the shaker was 100 RPM.

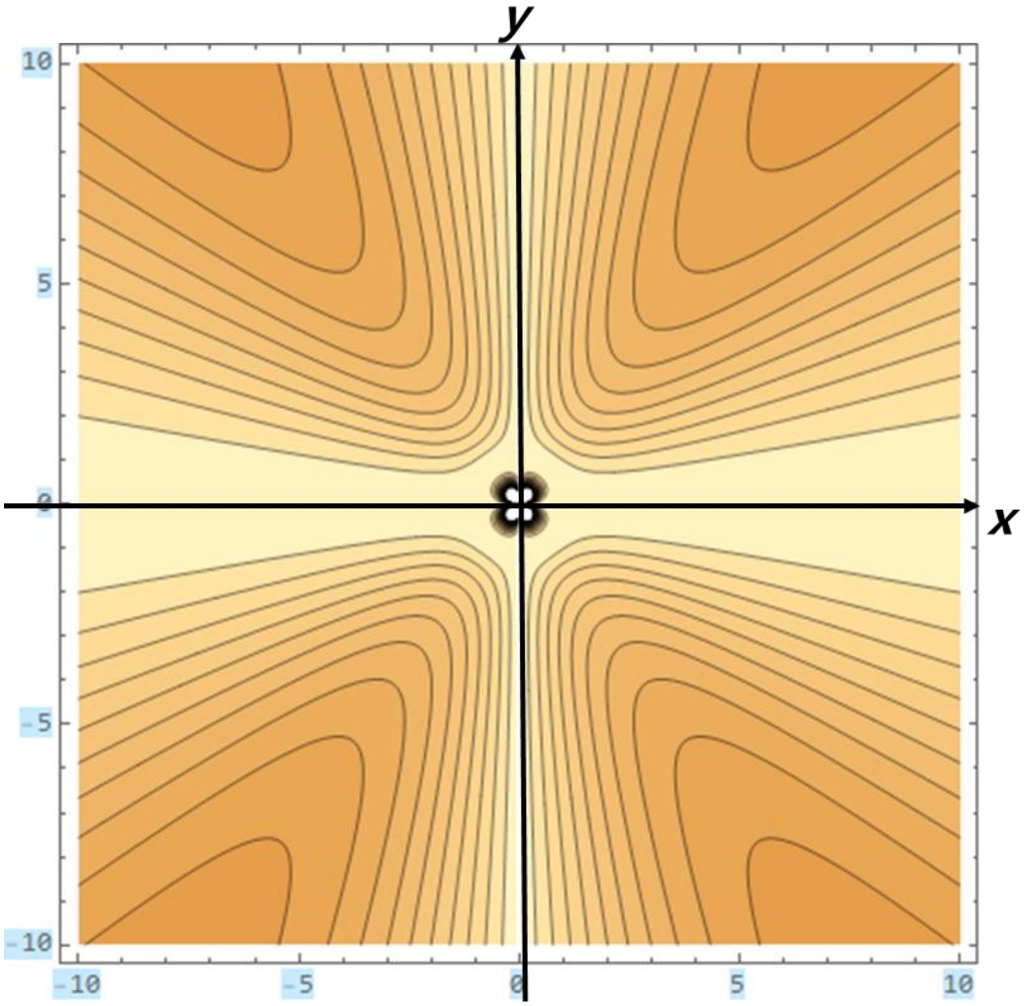

Figure S4. A contour plot of the creeping flow field around the rotating sphere in low Reynolds number when the radius of the sphere is 1 and its relative coordinates whose ranging from -10 to 10, on x,y-plane, respectively. The contour map was plotted based on Equation (1) by Mathematica.

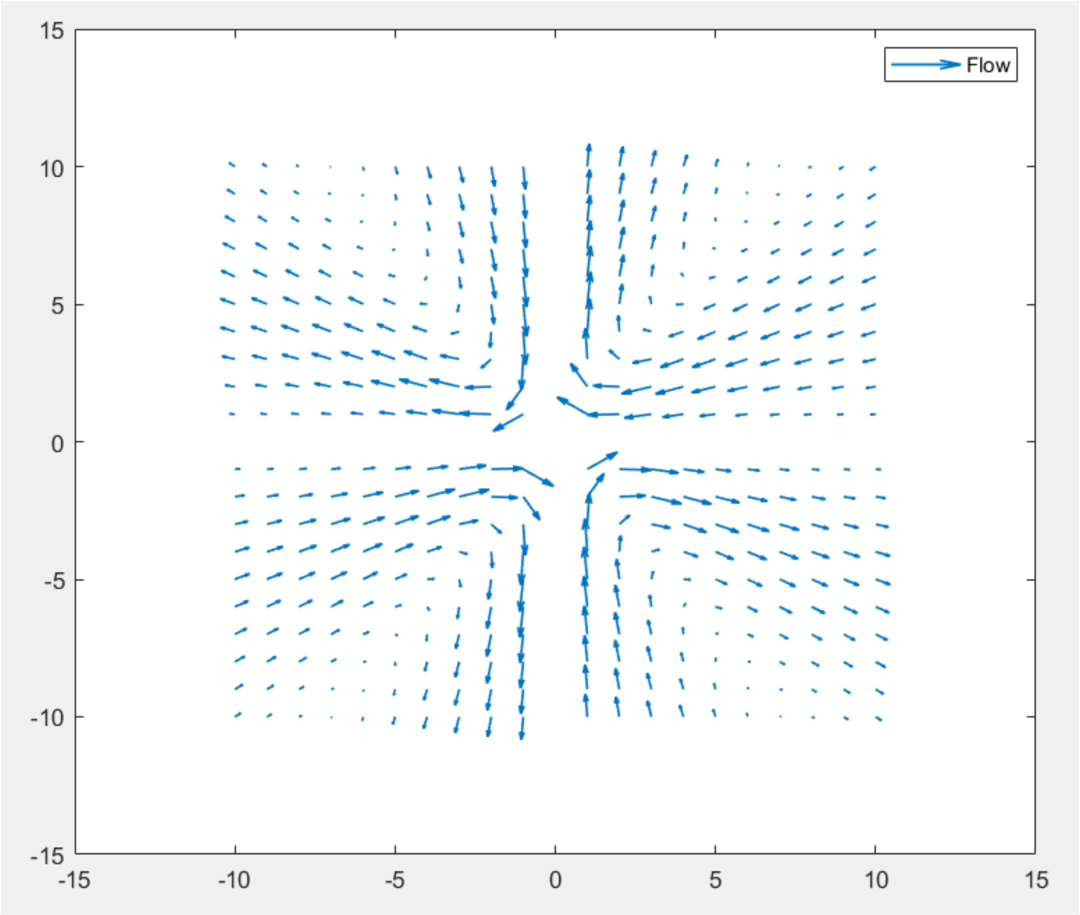

Figure S5. A visualized streamline of flow field around the rotating sphere. The streamline near the rotating sphere was plotted using the derivative of Equation (1) by MATLAB.
